# Development of Model Predictive Controller for a Tail-Sitter VTOL UAV in Hover Flight

**DOI:** 10.3390/s18092859

**Published:** 2018-08-30

**Authors:** Boyang Li, Weifeng Zhou, Jingxuan Sun, Chih-Yung Wen, Chih-Keng Chen

**Affiliations:** 1 Department of Mechanical Engineering, The Hong Kong Polytechnic University, Hong Kong, China; boyang.li@connect.polyu.hk (B.L.); chandler.zhou@connect.polyu.hk (W.Z.); jingxuan.j.sun@connect.polyu.hk (J.S.); cywen@polyu.edu.hk (C.-Y.W.); 2 Department of Vehicle Engineering, National Taipei University of Technology, Taipei 10608, Taiwan

**Keywords:** unmanned aerial vehicles (UAV), vertical takeoff and landing (VTOL), tail-sitter, model predictive control (MPC), hardware-in-loop (HIL) simulation, flight experiment

## Abstract

This paper presents a model predictive controller (MPC) for position control of a vertical take-off and landing (VTOL) tail-sitter unmanned aerial vehicle (UAV) in hover flight. A ‘cross’ configuration quad-rotor tail-sitter UAV is designed with the capabilities for both hover and high efficiency level flight. The six-degree-of-freedom (DOF) nonlinear dynamic model of the UAV is built based on aerodynamic data obtained from wind tunnel experiments. The model predictive position controller is then developed with the augmented linearized state-space model. Measured and unmeasured disturbance model are introduced into the modeling and optimization process to improve disturbance rejection ability. The MPC controller is first verified and tuned in the hardware-in-loop (HIL) simulation environment and then implemented in an on-board flight computer for real-time indoor experiments. The simulation and experimental results show that the proposed MPC position controller has good trajectory tracking performance and robust position holding capability under the conditions of prevailing and gusty winds.

## 1. Introduction

Small unmanned aerial vehicle (UAV) systems have gradually evolved from a commercial aerial photographic platform into industrial tools. They can conduct various kinds of missions, such as cargo delivery, highway inspection, and search and rescue. This trend has led to increased requirements for small UAV systems’ performance, including flight range, endurance, and payload capacity. Traditional airframe configurations such as fixed-wing, multi-rotor and helicopter UAVs are widely adopted. However, the shortcomings of each configuration limit many applications of UAV systems especially in high-density urban areas. Fixed-wing UAVs require runways or complicated catapults for take-off and landing which are not commonly available in most urban areas. The flight endurance and range for multi-rotor and helicopter UAVs are usually inadequate due to their low cruise efficiency.

Vertical take-off and landing (VTOL) UAVs combine the advantages of fixed-wing and rotary-wing UAVs. They can hover like traditional multi-rotors or helicopters and transition into level flight like fixed-wing aircrafts with high aerodynamic efficiency. Among different kinds of VTOL configurations, tail-sitter UAVs eliminate the complicated mechanism design and the consequent low reliability, but also introduce extra difficulties in the control system development due to large flight envelop and vulnerability to cross wind. Quad-rotor tail-sitter UAVs use a hover control method similar to that of ordinary quad-rotors with control moments mainly provided by the differential thrust of propellers. In addition, the traditional control surfaces on fixed wing aircrafts can also be used for hover and level flight control, which can provide plentiful and redundant control effort in all flight states. All of these features enable quad-rotor tail-sitters with a stable performance in hover flight because the control actuators are less likely to be affected by the outdoor environment.

Model predictive control (MPC) is a kind of optimal control approach that uses a receding horizon structure. It was developed in the 1970s and has been successfully used in process control, due to its capability of handling complex multi-input and multi-output systems with certain physical constraints [1]. The main idea of MPC is using the system dynamics to predict the system performance, and then deciding the optimal control actions by optimizing the predicted states. The finite time optimal control law is computed online by solving the optimization problem repeatedly. Another advantage of MPC is that it is able to optimize the system performance of the current time-slot while keeping that of future time-slots into account. Nowadays, with the improvement of computation power, the application of MPC gradually moves to the control of nonlinear and fast processes such as aerospace and robotic systems including UAVs [2].

The main contribution of this work is the development of the MPC position controller for a tail-sitter UAV in hover flight for trajectory tracking and disturbance rejection. To the best of our knowledge, no previous study has tried to use model predictive control on a tail-sitter UAV to improve the position control performance. Measured and unmeasured disturbances were introduced into the MPC model to improve the disturbance rejection ability of the UAV in a cross wind. The MPC controller was first simulated and then implemented in the on-board computer for real-time flight tests. The indoor trajectory tracking and hover experiments with MPC were compared with the traditional PID controllers.

The rest of this paper is organized as follows. Section 2 reviews related literature about design, modeling, and control of tail-sitters. Section 3 briefly describes the aircraft design and system configuration. Section 4 introduces the nonlinear dynamic model developed for simulation. The formulation of the MPC controller is presented in Section 5. The simulation and experimental results are given in Section 6 and Section 7, respectively. Conclusions are presented in Section 8.

## 2. Related Work

For tail-sitter design, the main factor to be determined is the number of thrust source. Single-propeller tail-sitter UAVs are more like a traditional fixed wing UAV. To keep the balance of momentum, we can use either two coaxial contra-rotation propellers [3] or multiple control surfaces to get enough aerodynamic momentum to balance the momentum from the propeller [4]. The advantage of single-propeller tail-sitters is the simple propulsion system, but the take-off-weight is rather limited. The dual-propeller configurations uses propellers combined with control surfaces submerged in the propeller slipstreams to produce control moments. In 1996, Stone et al. [5] developed a dual-rotor tail-sitter UAV named ‘Tandem-Wing’. Forshaw et al. [6] developed “QinetiQ’s Eye-On” with two helicopter rotor structures in 2011. Robin et al. [7,8] uses a fixed wing UAV called TBS Caipirinha and reconstructed it into a tilt-sitter in 2015. Verling et al. [9] developed a tail-sitter called ‘Pacflyer S100’ in 2016. Similar to single-propeller tail-sitters, the actuator efforts for dual-rotors in hover flight is easily disturbed by the cross or vertical wind. In the landing stage, the uncertain airflow passing the elevons often lead to out of control of UAVs so they must land with a very slow speed.

For quadrotor tail-sitters, Oosedo et al. [10] presented two quad-rotor tail-sitter UAV in 2011, one is cross type and the other is asterisk type. Hochstenbach and Notteboom [11] developed a tail-sitter called “VertiKUL” for parcel delivery. Lyu et al. [12] developed a quad-rotor tail-sitter based on a commercial airframe. They discussed the vibration attenuation method in the design process. Four-propeller tail-sitters use the similar control principle with quad-rotor in the hover flight. When transitting into level flight, they can either use all four or certain part of motors to provide thrust. They have the simple structure, stable control actuators, and high payload capability.

For the modeling methods of small UAVs, Knoebel et al. [13] built the aerodynamic model of a tail-sitter in hover condition. The propel, ailerons, elevator and rudder were modeled separately based on simplified low speed aerodynamic principles. Stone [14] predicted the aerodynamic forces on the vehicle using a full azimuthal base-element solution for the propellers combined with a panel method model. This modeling method can take the slipstream effects into consideration based on the propeller solution. Selig [15,16] did a large number of experiments to model the full-envelop aerodynamics of fixed wing UAVs. They considered high angles of attack (AoA) and high side-slip in maneuverer accomplished using large control surfaces. The author also used a component-based approach to model the vehicle in the full AoA and side-slip angle angle. Puopolo and Jacob [17] modeled the longitudinal perch maneuverer of a fixed-wing UAV. Perch is a motion that birds usually use under deep stall. This condition may also happen when the tail-sitter transit from hover to level flight. Zhang et al. [18] did full wind tunnel experiments to build the aerodynamic model. For the development of tail-sitters, the model of fixed-wing and multi-rotors cannot be built separately. In addition, the nonlinear model during transition stage were seldom discussed. Sun et al. [19] developed the hardware-in-loop simulation platform for a dual-rotor tail-sitter UAV based on the Simulink Desktop Real-Time Toolbox (R2017b, The MathWorks, Inc., Natick, MA, USA). The aerodynamic model was obtained from the wind tunnel experiments for the full AOA range.

In recent years, using the MPC to control UAVs attracted many researchers’ interests [20]. Kim et al. [21] investigated the feasibility of a nonlinear MPC in the control of unmanned helicopter in 2002. They concluded that the MPC demonstrates better tracking performance and robustness over traditional multi-loop PID controllers. In 2006, Slegers et al. [22] developed a nonlinear model predictive control strategy focused on aircrafts that can be adequately modeled with a rigid 6-DOF model. They simulated the applications to a parafoil, a payload aircraft, and a glider. Bemporad et al. [23] firstly proposed a hierarchical hybrid MPC for a quad-rotor in 2009. The linear MPC controller was designed to stabilize the vehicle near the commanded set-points while an upper control layer used a hybrid MPC controller with a slower sampling rate for obstacle avoidance. Kang and Hedrick [24] used a nonlinear MPC to design a high-level controller for a fixed-wing UAV to track desired path. They verified the controller performance with the HIL simulation.

The experimental results with MPCs increase in the 2010s with the development of on-board computation power. Abdolhosseini et al. [25] proposed an efficient MPC algorithm to control a quad-rotor with fewer prediction steps and less computation loads. Their simulation showed good tracking performance, but the experimental results were merely acceptable. Bouffard et al. [26] proposed a learning-based MPC for a quad-rotor. They tested the dynamic performance of the controller using ball-catching experiments. Alexis et al. [27] developed a switching MPC for a quad-rotor and conducted indoor experiments with hover. Bangura and Mahony [28] combined the hierarchical control paradigm and used the MPC as the outer loop controller for a quad-rotor in the indoor VICON environment (Vicon Motion Systems Ltd., Yarnton, Oxford, UK). The MPC has also been used to some novel UAV configurations such as quad-tiltrotors [29], tri-tiltrotors [30,31], and tri-ducted fan UAVs [32].

Despite these reference works for UAV control with MPC, none of them has applied it to the tail-sitters. The disturbance rejection capability, which is important to tail-sitters, has not been emphasized. The stable hover performance is crucial to tail-sitter UAVs in the take-off and landing stage. As the initial state of forward transition, the hover performance will also influence the quality of transition flight. The MPC can provide the optimal control solution based on the predicted response of the system with current states and future references. The MPC also has the ability to take account the measured and unmeasured disturbance during the optimization process to provide active rejection to the measurable disturbance.

## 3. UAV System Configuration

In this section, the detail configuration of the UAV system is given. The design of the tail-sitter UAV is introduced first. Then, the avionics and propulsion system are described.

### 3.1. Aircraft Design

**Figure 1:**
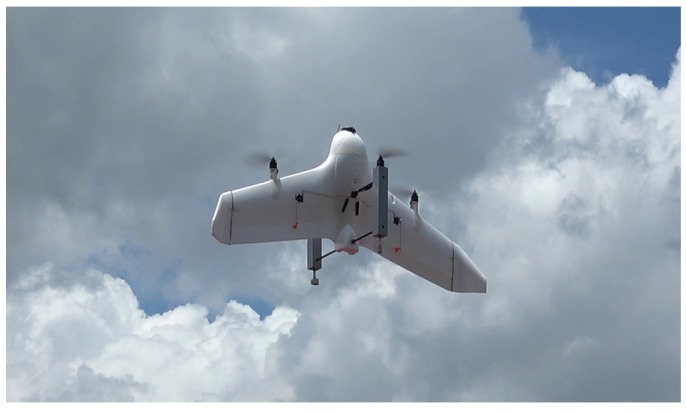
The prototype of the VTOL tail-sitter UAV in the air.

In this work, a quad-rotor tail-sitter UAV with ‘cross’ motor configuration is designed, as shown in Figure 1. The UAV was modified from a commercial flying wing UAV platform called ‘Skywalker X-5’. The wing span of the UAV is 1.1 m with a mean aerodynamic chord of 0.4 m and a take-off weight of 2.0 kg. The quad-rotor tail-sitter can use either a ‘×’ or ‘+’ configuration. We adopted the ‘+’ design since this configuration can utilize the original wing body of the vehicle as two of the motor bases to minimize the structural weight. Two 3D-printed motor seats are added to the leading edges of the wing on both sides. The downstream from these two propellers can improve the total airspeed on the main wing, which will increase the lift as well as improve the actuator effect of the elevons. The other two laser-cut wood motor seats are mounted on the upper and lower sides of the wing and connected by carbon tubes. In the normal flight condition, the differential thrust of four propellers can provide all control moments in three axes. The original control surfaces (elevons) with fixed wing airframe can also be utilized to provide extra control efforts in all flight stages including hover, transition, and level flight.

### 3.2. Avionics and Propulsion System

**Figure 2:**
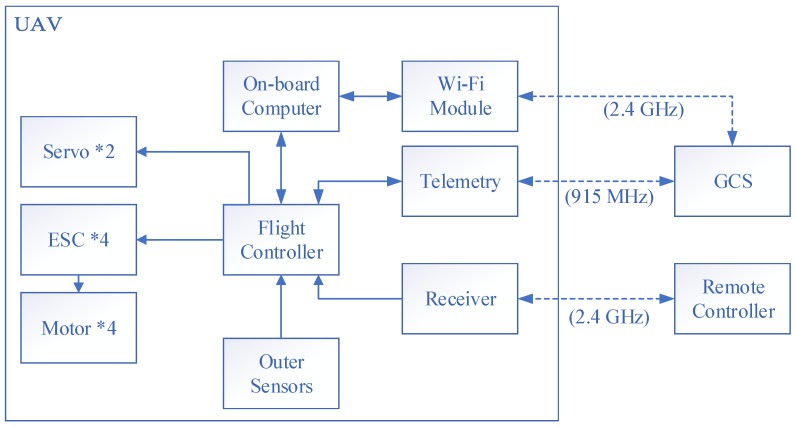
The avionics system of the UAV.

The avionics system of the UAV includes an on-board computer, a flight controller, actuators, sensors, a pair of telemetry, and a receiver. The Odroid XU4 (Hardkernel co., Ltd., Anyang, GyeongGi, Korea) is used as the on-board companion computer with the main Cortex™-A15@2Ghz CPU (Exynos5422, Samsung Semiconductor, Seoul, Korea). The powerful computation resource can satisfy the requirement of high-level control algorithm based on optimization such as MPC. Pixhawk and PX4 firmware [33] (v1.7.2) are used as the basic flight control unit. Pixhawk provides the basic attitude control and state estimation for the UAV while the higher level controllers will be deployed on the companion computer. The actuator system has four thrust sources with electronic speed controllers (ESC), motors, and propellers. Two digital servos driving elevons are also connected with the flight controller. The propulsion system is made up with 2212-980 kv motor, 30A ESC, and APC1147 propellers, providing a maximum thrust/weight ratio of 2. The power source of the system is a 4S 2600 mAh Li-Po battery. The Sik telemetry radio [34] was adopted for general bidirectional communication in our system. This pair of radio is small, light, and compatible with all serious Pixhawk flight controllers. The 915 MHz/500 mW version was selected according to the FCC standard. The on-board companion computer is connected with the ground control station (GCS) in a local area network by a 2.4 GHz Wi-Fi module for the off-board control management. A remote control and receiver system stands by for emergent situations. The summary block diagram of the avionics system is shown in Figure 2.

## 4. Dynamic Modeling

In this section, the six-degree-of-freedom (DOF) nonlinear dynamic model of the tail-sitter UAV is built for flight simulation and controller design. The simulation model of the UAV is important for the model-based controller design and performance analysis. A trustworthy model built from structural modeling method can significantly improve the design and testing efficiency. For a whole UAV model, several parts play important roles, including aerodynamic model, dynamic model, and actuator model.

### 4.1. Coordinate System

**Figure 3:**
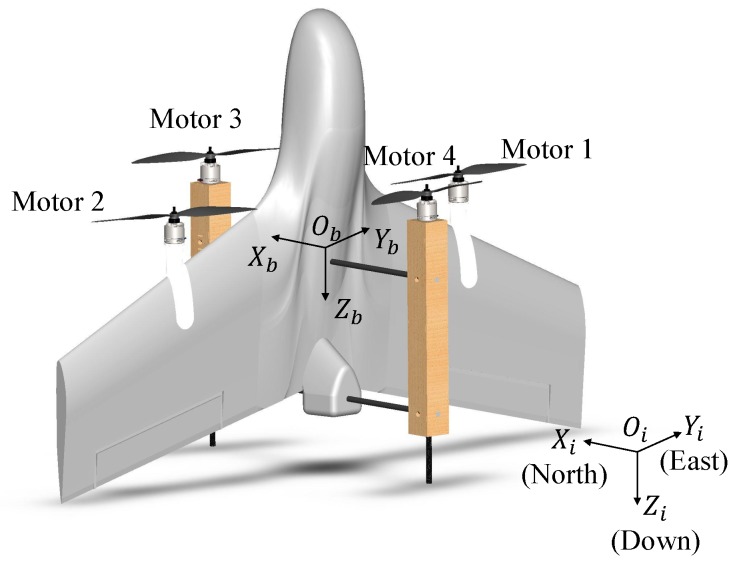
The inertial coordinate $\Gamma_{i}$ and body coordinate $\Gamma_{b}$ of the UAV.

Two coordination systems were used to describe the states of the UAV, as shown in Figure 3. The fixed inertial frame ($\Gamma_{i}:O_{i},X_{i},Y_{i},Z_{i}$) points to North-East-Down (NED) of the earth. The mobile body frame ($\Gamma_{b}:O_{b},X_{b},Y_{b},Z_{b}$) is located at the center of gravity (CG) of the UAV with the $X_{b}$-axis pointing to the front and the $Y_{b}$-axis to the right side of the main wing.

### 4.2. Dynamic Model of Vehicle

The position of the CG of the vehicle in $\Gamma_{i}$ is defined by $\xi ={\left[X\;Y\;Z\right]}^{T}\in R^{3}$, the velocity in $\Gamma_{i}$ is described by $v={\left[\dot{X}\;\dot{Y}\;\dot{Z}\right]}^{T}\in R^{3}$, the orientation of the vehicle is denoted by $R_{B}^{I}\in SO\left(3\right)$, which stands for 3D rotation group, and the angular velocity is denoted by ω. For an intuitive illustration, the desired and estimated attitudes are converted to Euler angles with ϕ,θ,ψ as roll, pitch, and yaw angles to describe the attitude of the tail-sitter in the later simulations and experiments. The kinematics and dynamics of the position and attitude can be described by [35]

(1) $$\begin{array}{cc} \dot{\xi } & =v,\\ m\dot{v} & =R_{B}^{I}F_{B},\\ {\dot{R}}_{B}^{I} & =R_{B}^{I}\omega_{\times },\\ I\dot{\omega } & =-\omega \times \left(I\omega \right)+M_{B}, \end{array}$$

where *m* denotes the mass and I the inertia matrix of the vehicle. $\omega_{\times }$ is the skew-symmetric matrix such that $\omega_{\times }v=\omega \times v$ for any vector $v\in R^{3}$. The $F_{B}$ and $M_{B}$ are total force and moment acting on $\Gamma_{b}$ respectively, which can be further divided as

(2) $$\begin{array}{cc} F_{B} & =F_{\mathrm{aero}}+F_{\mathrm{prop}}+R_{B}^{I}F_{g}+F_{d},\\ M_{B} & =M_{\mathrm{aero}}+M_{\mathrm{prop}}+M_{d}. \end{array}$$

$F_{\mathrm{aero}}$ and $M_{\mathrm{aero}}$ denote the aerodynamic force and moment vectors on the wing. $F_{g}$ is the gravity force. $F_{\mathrm{prop}}$ and $M_{\mathrm{prop}}$ are the thrust and moments created by the rotating propellers, respectively. $F_{d}$ and $M_{d}$ are the disturbance force and moment.

### 4.3. Aerodynamic Model

For tail-sitter UAVs, the wing suffers from the strong induced flow generated by the propellers. The aerodynamic effect for the vehicle must thus be taken into account in the modeling process. The flow condition for the part immersed in the induced flow should be different from the other wind part. In our model, the main wing is divided into five segments according to the diameter of the induced flow calculated by one-dimensional momentum theory. Here, we only took lift, drag and pitch moment into account and neglected side force and other moments. The details about the division process are shown in our previous work [36].

The considered force and moment generated by each segment were calculated separately according to the local flow condition and were summed up as the total aerodynamic term

(3) $$\begin{array}{cc} F_{\mathrm{aero}} & =L+D=\left[\begin{array}{c} -\sum L_{i}\cos\alpha_{i}\\ 0\\ -\sum L_{i}\sin\alpha_{i} \end{array}\right]+\left[\begin{array}{c} -\sum D_{i}\sin\alpha_{i}\\ 0\\ \sum D_{i}\cos\alpha_{i} \end{array}\right],\\ M_{\mathrm{aero}} & =\sum \left[\begin{array}{c} F_{\mathrm{aero},z}^{i}l_{y}^{i}\\ M_{i}+F_{\mathrm{aero},x}^{i}l_{z}^{i}\\ F_{\mathrm{aero},x}^{i}l_{y}^{i} \end{array}\right], \end{array}$$

where $\alpha_{i}$ denotes the local angle of attack of the *i* th segment. $L_{i}$, $D_{i}$, and $M_{i}$ are lift, drag force, and pitch moment, respectively. *l* denotes the lever of force between CG and the local aerodynamic center in the corresponding axes.

The lift, drag, and pitch moment on each segment can be further expressed as:

(4) $$\begin{array}{cc} L_{i} & =\frac{1}{2}\rho v_{i}^{2}S_{i}C_{L,i},\\ D_{i} & =\frac{1}{2}\rho v_{i}^{2}S_{i}C_{D,i},\\ M_{i} & =\frac{1}{2}C_{M,i}\rho v_{i}^{2}S_{i}{\bar{c}}_{i}, \end{array}$$

where ρ is the density of air, $S_{i}$ the area of the wing segment, and $v_{i}$ the local airspeed. $C_{L,i}$, $C_{D,i}$ and $C_{M,i}$ are the lift, drag, and moment coefficients, respectively, for the wing segment under the local flow condition.

There are several methods to get the aerodynamic coefficients for an aircraft, including computational fluid mechanics (CFD), wind tunnel experiments, and engineering estimation methods. For the small AOA under the stall angle (≈$20^{\circ }$), the aerodynamic coefficients usually can be obtained by linear approximation [37] or simplified CFD methods such as XFLR5 [38]. However, these methods cannot get satisfactory results at large AOA states. In this work, aerodynamic coefficients for the angle of attack from $0^{\circ }$ to $180^{\circ }$ are measured by experiments in a closed-wall wind tunnel with a test section of 600 mm × 600 mm × 2000 mm. The wing model is half-scaled due to the size of the test section. The wing model is fixed on a rotate plate by a force/moment sensor. The aerodynamic coefficients under different angles of attack are measured and finally built up to a look-up table in our previous work [39].

### 4.4. Actuator Model

**Figure 4:**
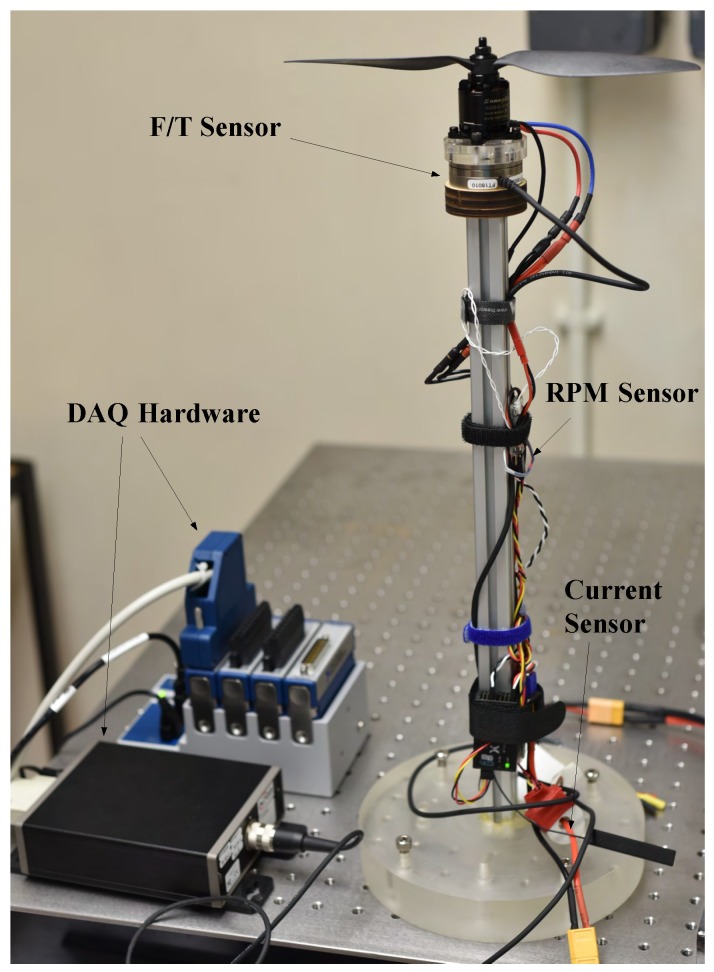
The set-up of propulsion system performance experiments.

The forces and moments generated by the propeller system at different rotation speeds were measured using static thrust experiments. The experimental configuration is shown in Figure 4. The propeller and motor were fixed on an ATI Mini-40 force/torque sensor and then connected to the fixed desk via an aluminum alloy beam. The rotation speed of the motor and propeller was controller by ESC and pulse-width modulation (PWM) signal generator. The actual rotation speed was measured by a FrSky Smart Port RPM (revolutions per minute) sensor (FrSky Electronic Co., Ltd., Wuxi, Jiangsu, China). The force and moments generated by the propulsion system at different throttle values were collected by the NI-9220 data acquisition (DAQ) system (National Instruments, Austin, TX, USA).

**Figure 5:**
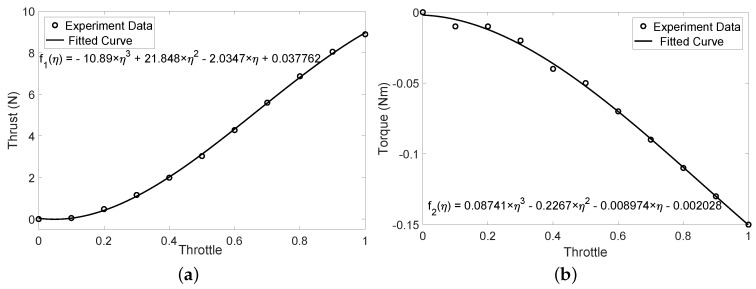
The experiment data and fitted curve of the motor-propeller system. (**a**) thrust to throttle; (**b**) torque to throttle.

The thrust and torque data were collected with throttle value ranging form 0 to 100%. At each throttle, we recorded the data for 5 s and then calculated the average value. The data were finally fitted with third-order polynomial curves. The original test points and fitted curves are shown in Figure 5.

## 5. Development of MPC Controllers

In this section, the formulation of the MPC controller is introduced. The controller structure is presented first, followed by the augmented prediction model and MPC algorithm formation. The dynamic model of UAV is linearized. Measured and unmeasured disturbance are included to improve the disturbance rejection performance.

### 5.1. Controller Structure

**Figure 6:**
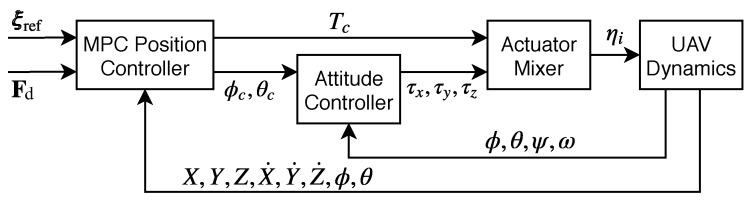
The cascaded two-loop control structure.

The controller is used with the structure scheme shown in Figure 6. It is assumed that all states of the vehicle are available from the on-board state estimator. The MPC is used as the outer-loop position controller and runs on the companion computer. The command attitude is handled by the inner-loop attitude controller and the actuator mixer running on the Pixhawk.

### 5.2. Augmented Prediction Model

Assuming a linearized, discrete-time state-space model of the plant, an *n* order, *r* input, *m* output system can be formulated as

(5) $$\begin{array}{c} x(k+1|k)=Ax(k|k)+\mathrm{Bu}(k|k),\\ y(k|k)=\mathrm{Cx}(k|k)+\mathrm{Du}(k|k), \end{array}$$

where the dimension of state matrix $A\in R^{n\times n}$, input matrix $B\in R^{n\times r}$, output matrix $C\in R^{m\times n}$, and direct transition matrix $D\in R^{m\times r}$. x is the system state vector and y is the system output vector. The suffix (k|k) means the prediction of future *k*-th step at the time step *k*. In the remaining parts of this work, we suppose D=0 and use (k) instead of (k|k) for conciseness if no confusion would happen.

The Bu(k) term can be further decomposed to

(6) $$\mathrm{Bu}\left(k\right)=B_{u}u_{u}\left(k\right)+B_{v}u_{v}\left(k\right)+B_{d}u_{d}\left(k\right).$$

Here, $u_{u}\left(k\right)$, $u_{v}\left(k\right)$, and $u_{d}\left(k\right)$ are the manipulated variables, measured disturbances, and unmeasured input disturbances, respectively. $B_{u}$, $B_{v}$, and $B_{d}$ are the corresponding columns of the control matrix B.

#### 5.2.1. Measured Disturbance

The controller can cancel the effect of some disturbances if they can be anticipated, known as feed forward. Feed forward can be incorporated into the predictive control by including the effects of the measured disturbances in the prediction model of future outputs. In the conventional control method, the controller reacts only after the system is affected by a disturbance and deviates from the reference value. While using the measured disturbance $u_{v}$ in Equation (6), the MPC controller can manage the compensation of $u_{u}$ as long as the disturbance is measured. This results in a faster reaction than a conventional feedback method. Since the measured disturbance value keeps varying according to the real time situation, control signal can be generated upon each disturbance magnitude at each time-slot.

The main disturbance source for the tail-sitter UAVs in hover flight is the cross wind. The vertical wing surface facing perpendicular to the wind will generate large force in the $X_{b}$-direction (see Figure 3). In our MPC controller, $F_{d,x}$ in Equation (2) is considered as the main disturbance force, which is assumed to be proportional to the magnitude of the cross wind. Currently, various methods are available to get the magnitude and direction of the disturbance wind by either direct measurement or estimation [40]. It is supposed that the wind information is available in the following simulations and experiments. The estimated disturbance force was fed into the prediction model of the MPC controller to compensate for the disturbance force in the $X_{b}$ direction.

#### 5.2.2. Unmeasured Disturbance

If there is a steady-state gain error between the real and prediction model, unmeasured disturbance can be used to reject unknown disturbances. The set-up of unmeasured disturbance requires an input disturbance model $u_{d}\left(k\right)$, which is a part of the MPC prediction model. The disturbance model influences the dynamic response when the measured plant output deviates from the desired trajectory due to an unknown disturbance or modeling error. The controller will continue to adjust until the plant output returns to its desired trajectory, like a classical integral feedback controller.

If the system state is augmented with integrating disturbance $u_{d}\left(k\right)$ driven by white noise $w_{d},$

(7) $$u_{d}\left(k+1\right)=u_{d}\left(k\right)+w_{d}.$$

The augmented system model can be further written as

(8) $$\begin{array}{cc} \left[\begin{array}{c} x(k+1)\\ u_{d}\left(k+1\right) \end{array}\right] & =\left[\begin{array}{cc} A & B_{d}\\ 0 & I \end{array}\right]\left[\begin{array}{c} x(k)\\ u_{d}\left(k\right) \end{array}\right]+\left[\begin{array}{cc} B_{u} & B_{v}\\ 0 & 0 \end{array}\right]\left[\begin{array}{c} u_{u}\left(k\right)\\ u_{v}\left(k\right) \end{array}\right]+w_{d},\\ y(k) & =\left[\begin{array}{cc} C & I \end{array}\right]\left[\begin{array}{c} x(k)\\ u_{d}\left(k\right) \end{array}\right]. \end{array}$$

**Figure 7:**
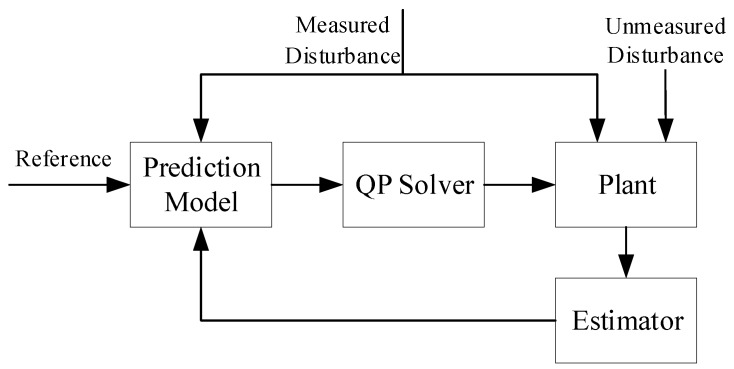
The MPC structure with the augmented system model.

$B_{d}$ can be chosen to present the effect of integrating disturbance to the states and measured outputs. Assuming the augmented system is observable, a standard linear observer can be employed to estimate the sate and disturbance vectors:

(9) $$\begin{array}{cc} \left[\begin{array}{c} \hat{x}\left(k+1\right)\\ {\hat{u}}_{d}\left(k+1\right) \end{array}\right]= & \left[\begin{array}{cc} A & B_{d}\\ 0 & I \end{array}\right]\left[\begin{array}{c} \hat{x}\left(k\right)\\ {\hat{u}}_{d}\left(k\right) \end{array}\right]+\left[\begin{array}{cc} B_{u} & B_{v}\\ 0 & 0 \end{array}\right]\left[\begin{array}{c} u_{u}\left(k\right)\\ u_{v}\left(k\right) \end{array}\right]\\ & -\left[\begin{array}{c} L_{x}\\ L_{d} \end{array}\right]\left(C\hat{x}\left(k\right)+{\hat{u}}_{d}\left(k\right)-y\left(k\right)\right), \end{array}$$

where $L_{x}$ and $L_{d}$ are estimator gains. The final augmented model and the relation with MPC controller is shown in Figure 7. In the tail-sitter UAV, there is an aerodynamic effect generated by the downstream of propellers, which leads to lift and drag. These disturbance are changing according to the rotation speed of the propellers. Thus, $F_{aero,x}$ and $F_{aero,z}$ are included in the prediction model as unmeasured disturbance to ensure the offset-free tracking.

### 5.3. Model Linearization

We adopted a cascaded control structure and assumed that the attitude of the vehicle is controlled by an inner loop attitude controller. The outer loop is the position controller which has the state and input as $x={\left[X\;Y\;Z\;\dot{X}\;\dot{Y}\;\dot{Z}\;\phi^{I}\;\theta^{I}\right]}^{T},u_{u}={\left[\phi_{c}^{I}\;\theta_{c}^{I}\;T_{c}\right]}^{T},u_{v}={\left[F_{d,x}\right]}^{T},u_{d}={\left[F_{aero,x}\;F_{aero,z}\right]}^{T}$ follows the definition in the prediction model. $T_{c}$ is the commanded thrust. $\phi_{c}^{I}$,$\theta_{c}^{I}$ are the roll and pitch angles in the inertial frame to get rid of the heading (yaw) angle ψ in the model. The transformation between the angle in the body frame and the inertial frame is

(10) $$\left[\begin{array}{c} \phi \\ \theta \end{array}\right]=\left[\begin{array}{cc} \cos\psi & \sin\psi \\ -\sin\psi & \cos\psi \end{array}\right]\left[\begin{array}{c} \phi^{I}\\ \theta^{I} \end{array}\right].$$

To achieve accurate trajectory tracking, the inner loop system dynamic should be considered. In this work, the inner loop attitude dynamic are approximated by a set of first-order transfer functions [41]

(11) $$\begin{array}{c} \dot{\phi }=\left(1/\tau_{\phi }\right)\left(\phi_{c}-\phi \right),\\ \dot{\theta }=\left(1/\tau_{\theta }\right)\left(\theta_{c}-\theta \right), \end{array}$$

where $\tau_{\phi }$, $\tau_{\theta }$ are the time constants of the roll and pitch angle control, respectively. Assuming that the deviation of the vehicle to the steady state is small in the hover flight, the dynamic and kinematic models can be linearized into the continues form of Equation (5), with the parameters

(12) $$A=\left[\begin{array}{ccc} 0_{3\times 3} & I_{3\times 3} & 0_{3\times 2}\\ 0_{3\times 3} & 0_{3\times 3} & \begin{array}{cc} 0 & g\\ g & 0\\ 0 & 0 \end{array}\\ 0_{2\times 3} & 0_{2\times 3} & \begin{array}{cc} -\tau_{\phi }^{-1} & 0\\ 0 & -\tau_{\theta }^{-1} \end{array} \end{array}\right],\;B=\left[\begin{array}{cccccc} & 0_{3\times 3} & & & 0_{3\times 3} & \\ 0 & 0 & 0 & \frac{1}{m} & \frac{1}{m} & 0\\ 0 & 0 & 0 & 0 & 0 & 0\\ 0 & 0 & \frac{1}{m} & 0 & 0 & \frac{1}{m}\\ \frac{1}{\tau_{\phi }} & 0 & 0 & 0 & 0 & 0\\ 0 & \frac{1}{\tau_{\theta }} & 0 & 0 & 0 & 0 \end{array}\right].$$

### 5.4. Objective Function

The objective function for the MPC controller is in a quadratic form that is formulated by the squares of the weighted state errors and the changing value of the control inputs

(13) $$J\left(z_{k}\right)=\sum_{i=0}^{H_{p}-1}\left\{\left[e_{y}^{T}\left(k+i\right)Qe_{y}\left(k+i\right)\right]+\left[e_{u}^{T}\left(k+i\right)R_{u}e_{u}\left(k+i\right)\right]+\left[\Delta u^{T}\left(k+i\right)R_{\Delta u}\Delta u\left(k+i\right)\right]\right\}.$$

Here, Q, $R_{u}$, and $R_{\Delta u}$ are positive semi-definite weight matrices. At the current control interval *k*, the cost function will consider a prediction horizon $H_{p}$, which consists of *i* steps. $e_{y}$, $e_{u}$ and Δu represent errors in output, input, and the change of input, respectively. They can be expressed as:

(14) $$\begin{array}{c} e_{y}\left(k+i\right)=S_{y}^{-1}\left[w\left(k+i+1\right)-y\left(k+i+1\right)\right],\\ e_{u}\left(k+i\right)=S_{u}^{-1}\left[u_{\mathrm{target}}\left(k+i\right)-u\left(k+i\right)\right],\\ \Delta u\left(k+i\right)=S_{u}^{-1}\left[u\left(k+i\right)-u\left(k+i-1\right)\right], \end{array}$$

where w(k+1) is the plant output reference values and y(k+1) is the plant outputs at the *i*-th prediction horizon step, u(k+i) is the input value of the *i*-th prediction horizon step at the *k*-th control interval, $u_{\mathrm{target}}\left(k+i\right)$ is the MV target corresponding to u(k+i), $S_{y}$ and $S_{u}$ are diagonal matrices of scale factor for plant output and input in engineering units.

One of the most significant characteristics of MPC is its ability to take account of constraints during optimization. Bounds constraints were added to the plant outputs and control inputs such that the MPC constraints can be expressed as follows:

(15) $$\begin{array}{c} y_{j,min}\left(i\right)\leq y_{j}\left(k+i\right)\leq y_{j,max}\left(i\right),\;i=1:H_{p},j=1:N_{y},\\ u_{j,min}\left(i\right)\leq u_{j}\left(k+i-1\right)\leq u_{j,max}\left(i\right),\;i=1:H_{p},j=1:N_{u},\\ \Delta u_{j,min}\left(i\right)\leq \Delta u_{j}\left(k+i-1\right)\leq \Delta u_{j,max}\left(i\right),\;i=1:H_{P},j=1:N_{u}, \end{array}$$

where $N_{y}$ and $N_{u}$ are the numbers of plant output and input. $y_{j,min}\left(i\right)$ and $y_{j,max}\left(i\right)$ are lower and upper bounds for the *j*-th plant output at the *i*-th prediction horizon step. $u_{j,min}\left(i\right)$, $u_{j,max}\left(i\right)$, $\Delta u_{j,min}\left(i\right)$ and $\Delta u_{j,max}\left(i\right)$ are having similar meaning but for MV and MV increment.

The cost function in Equation (13) and constraints in Equation (14) form a standard quadratic programming (QP) problem and it can then be solved by a QP solver [42]. $z_{k}^{*}$ is the QP decision given by

(16) $$z_{k}^{*}={\left[u{\left(k|k\right)}^{T}\;\;u{\left(k+1|k\right)}^{T}\ldots \;u{\left(k+H_{p}-1|k\right)}^{T}\right]}^{T},$$

in which only the $u{\left(k|k\right)}^{T}$ will be implemented at each time step. Several methods are available to solve the QP problems, such as Active Set methods, interior point methods, etc. In MATLAB, the KWIK algorithm will be used for QP solving [43].

## 6. HIL Simulation

To validate the real-time performance of the controller as well as to facilitate the tuning process, an HIL simulation environment was developed for the tail-sitter UAV. The structure of the HIL environment is described first. The final MPC parameters and simulation results are shown later.

### 6.1. HIL Simulation Structure

The computation cost of MPC controller is usually high since the optimization problem needs to be solved online. Therefore, an HIL simulation environment is important to test the real-time performance of the MPC controller. The test platform is made up of two parts, one is the nonlinear tail-sitter dynamic model running real-time in a desktop PC with an Intel Core i7-4790 CPU @ 3.60 GHz (Intel Corporation, Santa Clara, CA, USA), and the other is the MPC position controller running on the flight computer Odroid XU4 (Hardkernel co., Ltd., Anyang, GyeongGi, Korea). Odroid XU4 is a small (83 × 58 × 20 mm), light (38 g), and powerful on-board computer. It can run an Ubuntu 16.04 operation system, which is supported by ROS Kinetic Kame distribution [44]. Based on our experience, this board can run our generated MPC controller in real time.

**Figure 8:**
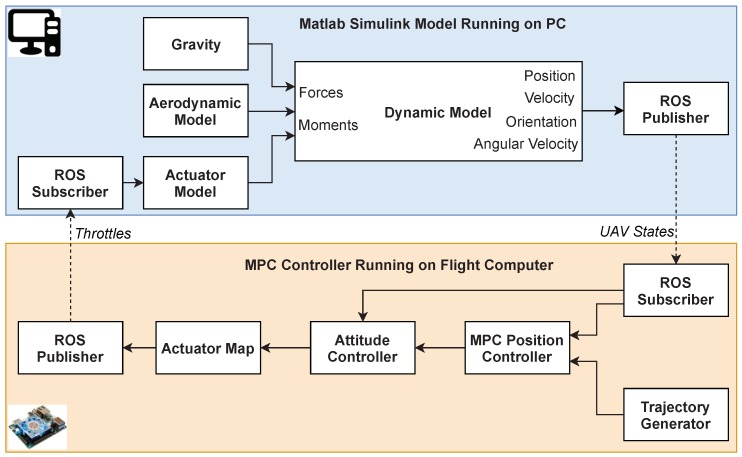
The structure of the HIL simulation environment.

Every part of the dynamic model in Section 4 were built in MATLAB Simulink. A third-party tool named Real-Time Pacer [45] was used to synchronize the simulation with wall clock time. The MPC controller describe in Section 5 was implemented by MATLAB and Simulink MPC Control Toolbox and then generated into C/C++ code in a ROS node by by the Simulink Coder. The generated code can be built and executed in the on-board computer with high efficiency. The two computers were connected with a router by a RJ-45 port and used ROS to exchange messages in the subscribing and publishing manner. The whole HIL simulation structure is shown in Figure 8.

### 6.2. Controller Parameters

The MPC controller has some parameters to be decided for the balance between tracking performance and robustness. The HIL simulation environment provides an efficient platform to tune these parameters. The first set of values to be decided are the prediction horizon $H_{p}$ and sampling time $T_{s}$. A small $T_{s}$ and a large $H_{p}$ usually improve the rejection of unknown disturbances but will increase the computational effort. In our MPC controller, the sampling time was selected as $T_{s}=0.05$ s and the prediction horizon $H_{p}=20$. The control horizon means the number of changeable control outputs to be considered in the QP solution in each control interval. $H_{u}=5$ was chosen for the balance of performance and computational load.

Adjusting the weights of variables is also critical to the performance. The different weights for output and control variables were used to balance the tracking speed and the cost of control effort. A higher weight of the output variable weight Q means a higher priority to eliminate the tracking error; while high weights of control output $R_{u}$ and control output rate $R_{\Delta u}$ mean high penalties to the absolute value and change of control effort.

The key parameters of the MPC controller used for this tail-sitter vehicle are shown in Table 1.

### 6.3. Simulation Results

**Figure 9:**
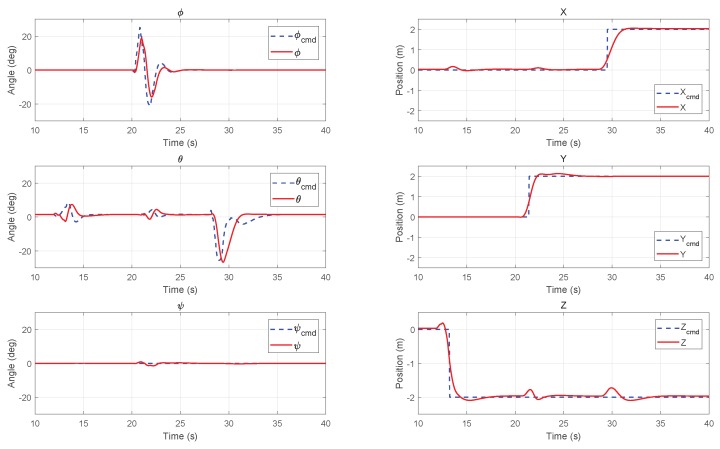
Step path tracking results of attitude (**left column**) and position (**right column**) in HIL simulation.

**Figure 10:**
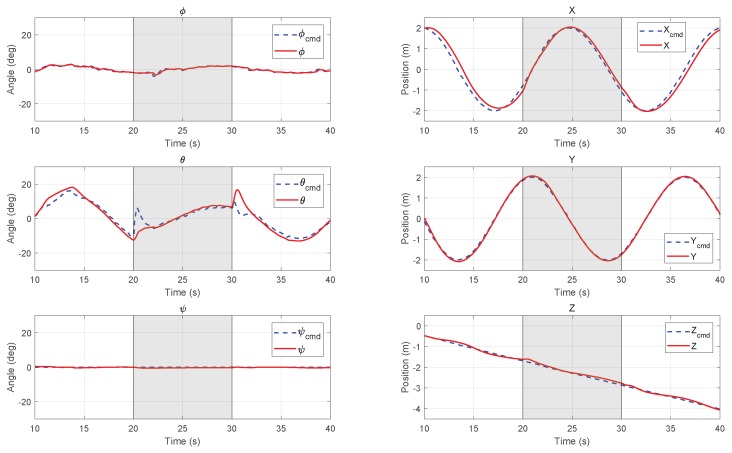
Inclining spiral path tracking results of attitude (**left column**) and position (**right column**) with wind disturbance from 20–30 s in the HIL simulation.

Two typical trajectory tracking simulations were carried out in the HIL simulation environment. The initial condition is the hover state at the origin point. The reference trajectories in $X_{i},Y_{i},Z_{i}$ directions were given to the MPC controller by a trajectory generator in advance. In the following Figure 9 and Figure 10, the commanded reference are plotted in dashed lines and the position and attitude responses of the UAV system are plotted in solid lines. The left column shows the roll, pitch, and yaw angles of attitude, and the right column shows the position in inertial frame.

#### 6.3.1. Step Path Tracking

Figure 9 shows the simulation results of the step path tracking. The step commands of 2 m in three directions ($Z_{i},Y_{i},X_{i}$) were given to the UAV in 13 s, 22 s, and 26 s, accordingly. It is shown that the vehicle can reach the desire position within 3 s with small overshoot. The commanded attitude was consistently constrained by MPC controller’s outputs, which is $\pm 30^{\circ }$ in roll and pitch angle. Some oscillations can be find in the pitch direction for 12–15 s and 21–23 s before the commanded translation on the *X*-axis. These oscillations were caused by the aerodynamic coupling of large movement in the other *Y* and *Z* directions.

#### 6.3.2. Circular Path Tracking with Wind

Figure 10 shows the simulation results of inclining spiral path tracking with artificially added wind of 2 m/s from 20–30 s during flight. In the simulation model, the wind effect was added by adding a horizontal speed to the flow condition calculation module. For the MPC controller, the measured disturbance input was also provided with an estimated force by the aerodynamic model during the same period. We can find that the vehicle can track the trajectory very well. When the wind starts and stops, the outer loop controller will generate the corresponding attitude target to resist wind and keep a small position tracking error.

## 7. Flight Experiments

The previous simulation environment verifies the MPC controller’s performance and provides its basic parameters. In this section, the MPC controller is further implemented in the on-board computer and connected with the flight controller to test the flight performance in the indoor environment.

### 7.1. Experiment Environment

**Figure 11:**
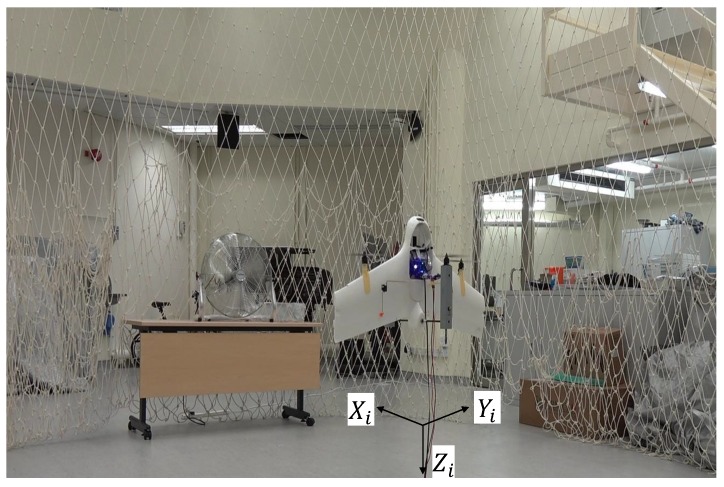
Indoor experimental environment with a VICON motion capture system on the ceiling.

The on-board MPC controller is a standard ROS node with C/C++ codes generated by Matlab Simulink Coder. This is an efficient method for quick evaluation of the MPC controller developed in the Simulink environment. The MAVROS [46] was used to exchange the states of the vehicle and the target attitude between the companion computer and the flight controller in the off-board control mode. The indoor position of the vehicle was detected by a VICON motion capture system (Vicon Motion Systems Ltd., Yarnton, Oxford, UK). The experimental environment is shown in Figure 11. The vehicle was powered by a line from the DC power supply. The external disturbance was simulated by an industrial fan blowing to the main wing of the vehicle in the $X_{i}$ direction.

**Figure 12:**
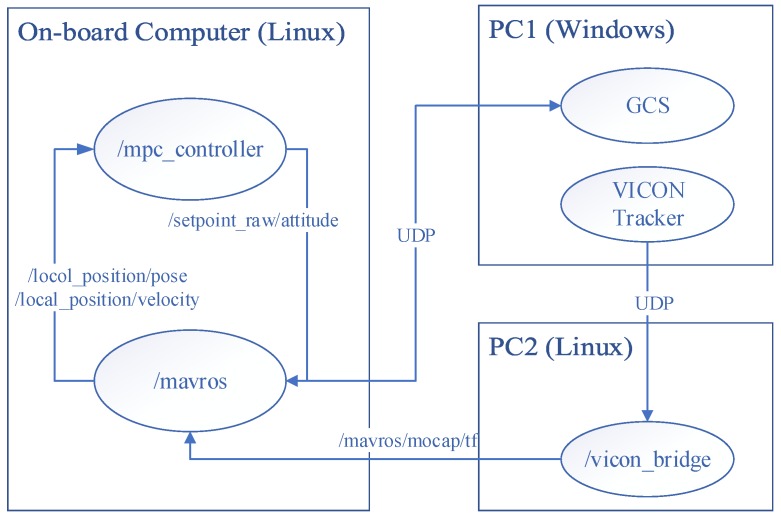
The communication network of the indoor experimental environment.

Similar with the HIL simulation platform, the communication in the experimental environment also adopted the ROS network for data exchange among different devices connected with a Wi-Fi router. The network structure with device names, ROS node names, and ROS message names is shown in Figure 12. PC1 is a desktop which runs the VICON data acquisition software and GCS for general flight state monitoring. The VICON software Tracker will send the local position of the UAV to PC2 which runs the ROS master and ‘vicon_bridge’ package [47]. The PC2 will then pass the position information to the MAVROS node running on the on-board computer Odroid XU4.

The local position will then be provided to the local position estimator (LPE) running on the Pixhawk and the final estimated states are published to the MPC controller node running on an on-board computer. The calculated control outputs, thrust set-point and attitude set-points are finally given to the flight controller via MAVROS in the off-board control mode.

### 7.2. Experimental Results

The experimental results show the path tracking and disturbance rejection ability of the MPC controller. The hover control experiments facing wind were conducted first. The original outer-loop PID controller from the open source platform is used as baseline controller for comparison with MPC controller. The circular trajectory tracking experiment was followed to show the tracking performance of proposed MPC controller.

#### 7.2.1. Hover with Wind Disturbance

**Figure 13:**
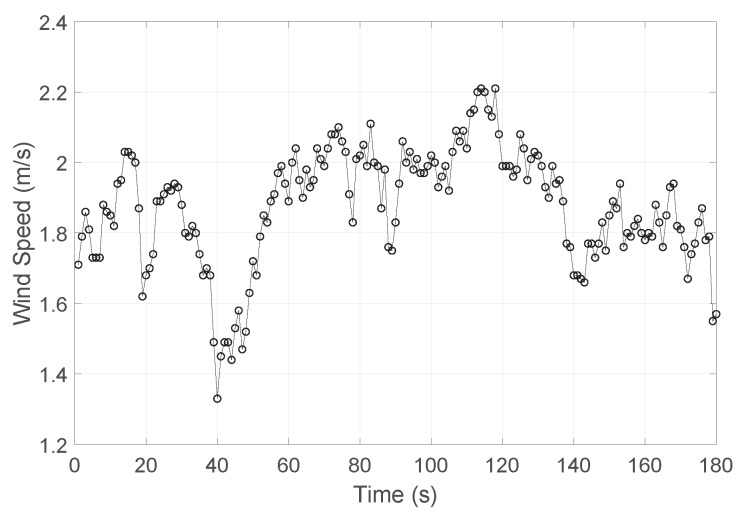
The non-directional wind speed of an industrial fan for three minutes.

One of the challenges for tail-sitter vehicles is the ability to resist cross wind in hover flight. As there will be a large wing surface facing wind vertically, the extra forces and moments caused by wind usually lead the vehicle to drift with wind. The hover flight tests with wind disturbance was conducted to evaluate the position holding performance of the MPC controller. In the indoor experiment, an industrial fan was used to simulate the cross wind blowing to the main wing of the vehicle. A digital air flow sensor, Testo-480 (Testo SE & Co. KGaA, Lenzkirch, Germany) with a Ø3 mm hot ball thermal flow velocity probe, was used to measure the flow speed from the fan at the proposed UAV hover position. The measured results for a period of three minutes is shown in Figure 13 with 1 Hz sampling rate. The calculated average wind speed is 1.9 m/s. Notably, the wind from the fan is vary unstable in magnitude.

**Figure 14:**
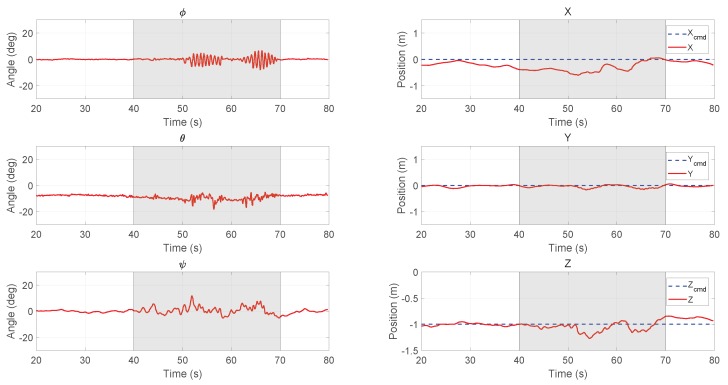
Experimental results of attitude (**left column**) and position (**right column**) for hovering with the PID controller (the fan was turned on for 40–70 s).

The ordinary PID controller provided by the PX4 firmware was first tested. The position tracking results are shown in Figure 14. The cascaded two-loop PID controller was firstly tuned with the Ziegler–Nichols method into an acceptable region to assure the basic movement of the vehicle. Then, the parameters were further fine-tuned manually based on experience. The position set-point for the vehicle was the origin of world coordinate at a height of 1 m. Before the wind started, there existed a tracking error in the *X*-direction. The error gradually reduced to zero but enlarged later. For tail-sitters, the trimmed pitch angle in hover state should not be zero since there will be a lift generated on the wing by the induced flow. If the PID controller gives a zero pitch command when there is no error in the *X* position, the vehicle will drift away immediately. From *t* = 40–70 s, a cross wind from aforementioned fan was blown toward the vehicle. When the wind hit the vehicle, the vehicle moved further away from the set-point for about 0.6 m downstream at maximum, and then gradually flew back in about 20 s. The root mean square errors (RMSE) for X,Y, and *Z* are 0.293 m, 0.061 m, and 0.090 m, respectively.

**Figure 15:**
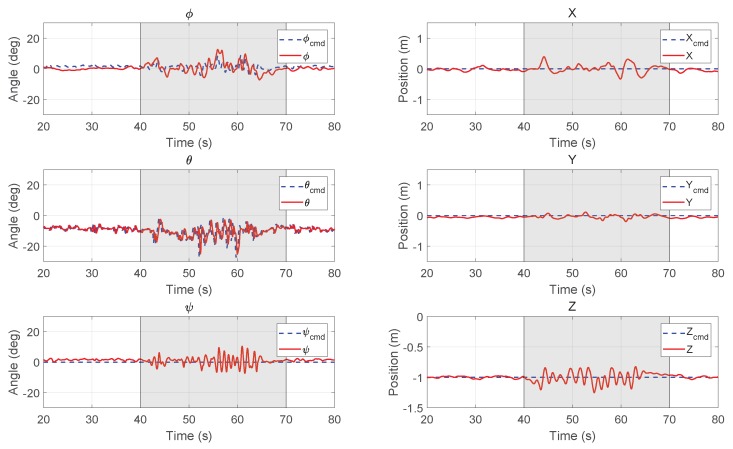
Experimental results of attitude (**left column**) and position (**right column**) for hovering with the MPC controller (the fan was turned on for 40–70 s).

Figure 15 shows the hover results with the MPC controller. As the wind disturbance from the fan kept changing, only unmeasured disturbance was added to the prediction model in the following MPC experiments. Compared with the results of the PID controller, we can find the tracking error is much smaller on the *X*-axis. There is also no obvious divergence during the wind disturbance. It is shown that the vehicle can tightly track the commanded trajectory in the cross wind. The small oscillation on each axis are mainly caused by the unstable flow from the fans shown in Figure 13. The RMSE for X,Y, and *Z* are 0.085 m, 0.057 m, and 0.080 m, respectively, which are smaller than the results for PID controller, especially in the *X* direction.

#### 7.2.2. Circular Trajectory Following

**Figure 16:**
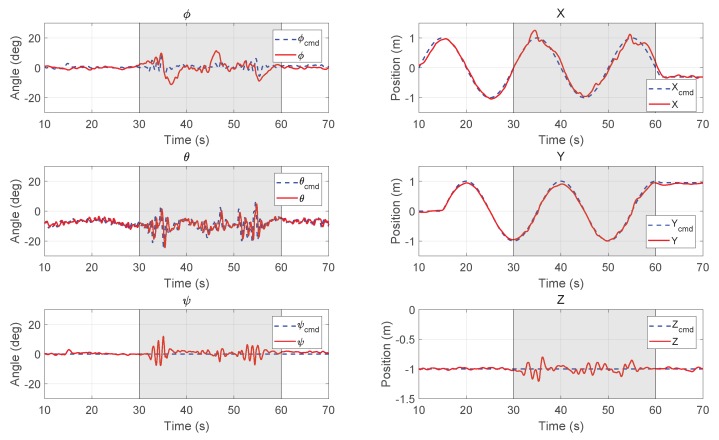
Experimental results of attitude (**left column**) and position (**right column**) for the UAV to follow a circular path with the MPC controller (the fan was turned on for 30–60 s).

When the hovering performance was verified, a circular trajectory was also given to the vehicle for experiments. The results are shown in Figure 16. It is shown that the MPC controller can guide the UAV to fly along the commanded path closely during the whole period. When the fan was turned on at t=30 s, the oscillation in pitch direction increased to resist the changing disturbance, while the tracking performance was not compromised. When the vehicle reached X=1 m (see Figure 11), the local wind has a largest mean value of 2.21 m/s, which caused a larger error than the downwind positions, where X=−1 m.

## 8. Conclusions

In this work, a tail-sitter VTOL UAV was designed and manufactured with both hovering and level fight capability. The 6-DOF nonlinear dynamic model of the vehicle was built for the controller development and simulations. A model predictive position controller was developed based on the augmented state-space model in hover flight. An HIL simulation environment was introduced based on the ROS communication network. The MPC controller is first evaluated by the HIL simulation and then implemented in the on-board flight computer for indoor flight tests. The results of hovering and trajectory tracking show that the proposed MPC controller demonstrates a good tracking performance and a disturbance rejection ability.

Future work will include the development of on-board wind measurement or estimation methods to provide more accurate and time-variant information about the measured disturbance to the MPC controller. Then, the outdoor experiments can be carried out in the natural prevailing and gusty wind environment. The optimal transition control strategy with MPC controller could also be studied in order to further improve the whole mission performance with such a model-based controller.

## Figures and Tables

**Table 1:** Parameter setting of MPC controller.

Parameter	Value
$T_{s}$	0.05
$H_{p}$	40
$H_{c}$	5
|y| constraint	${\left[\inf,\inf,\inf,20,20,20,\pi /6,\pi /6\right]}^{T}$
|y| scale factor	I
|u| constraint	${\left[\pi /6,\pi /6,10\right]}^{T}$
|u| scale factor	${\left[1,1,15\right]}^{T}$
Q	diag{8,8,5,5,2,2,10,8}
$R_{u}$	diag{0,0,0}
$R_{\Delta u}$	diag{10,4,10}

## Supplementary Materials

The following are available online at https://youtu.be/h6mhPSNC5Vo, Video S1: Indoor hover and position tracking experiments. https://youtu.be/kYevywwGgjQ, Video S2: Whole mission outdoor flight tests.

## Abbreviations

The following abbreviations are used in this manuscript:

AOA	angle of attack
CFD	computational fluid dynamics
CG	center of gravity
DAQ	data acquisition
DOF	degree of freedom
EOM	equations of motion
ESC	electric speed controller
FCC	federal communications commission
HIL	hardware-in-the-loop
LPE	local position estimator
LQR	linear quadratic regulation
MAC	mean aerodynamic chord
MD	measured disturbance
MPC	model predictive control
NED	north-east-down
PPT	PolyU Plus Tail-sitter
QP	quadratic-programming
RMSE	root mean square error
UD	unmeasured disturbance
UAV	unmanned aerial vehicle
VLM	vortex lattice method
VTOL	vertical takeoff and landing
